# Development of Au nanowire injector system to deliver plasmid into mouse embryo

**DOI:** 10.1016/j.dib.2017.07.017

**Published:** 2017-07-15

**Authors:** Kkotchorong Park, Keun Cheon Kim, Hyoban Lee, Yoori Sung, Mijeong Kang, Yun Mi Lee, Ji Yeon Ahn, Jeong Mook Lim, Taejoon Kang, Bongsoo Kim, Eun Ju Lee

**Affiliations:** a Department of Chemistry, KAIST, Daejeon 34141, South Korea; b Biomedical Research Institute, Seoul National University Hospital, Seoul 03080, South Korea; c Biomedical Research Center, KAIST, Daejeon 34141, South Korea; d Department of Agricultural Biotechnology, Seoul National University, Seoul 08826, South Korea; e Hazards Monitoring Bionano Research Center and BioNano Health Guard Research Center, KRIBB, Daejeon 34141, South Korea; f Major of Nanobiotechnology, KRIBB School, UST, Daejeon 34113, South Korea

**Keywords:** Gene delivery, Gold nanowire, Mosaicism, Nanoinjector, Transgenic animal

## Abstract

In this data article, we developed a Au nanowire injector (Au NWI) for directly delivering plasmid into the 1-cell stage of the mouse embryos designed to successfully attach and detach the plasmid on the Au NWI, highly minimizing physical and chemical damage on the embryos. This data presents that a Au NWI system does not induce detrimental damages on development of embryos and efficiently express the green fluorescence protein in vitro. The data provided herein is in association with the research article related to reduce the occurrence of mosaicism by a Au NWI,” Suppressing Mosaicism by Au Nanowire Injector-driven Direct Delivery of Plasmids into Mouse Embryos” (Park et al., 2017 [1]).

**Specifications table**

**Table:** 

Subject area	*Chemistry*
More specific subject area	*Gene delivery*
Type of data	*Scheme, Table, Image, EDAX spectra, Figure*
How data was acquired	*Microscope, Confocal microscope, Electrochemical Analysis, Energy dispersive X-ray analysis, qPCR*
Data format	*Analyzed, Raw*
Experimental factors	Synthesis of a Au NW and fabrication of a Au NWI were reported in previous study [1], [2] respectively. Mouse embryo were prepared as reported in a previous study [1].
Experimental features	*Electro-triggered detachment of plasmid: CHI 660D (CH instrument, USA), EDAX analysis and SEM imaging:* FEI Nova 230 (EI Company, USA), *qPCR analysis:* ABI Prism 7000 sequence detection system (Applied Biosystems, USA) with SYBR Green PCR Master Mix (4309155; Applied Biosystems, USA). *Au NWI injection:* conventional microinjection system (Sutter Instruments, USA) mounted on a Leica micromanipulator, *Statistical analysis:* GraphPad Prism 5 (GraphPad Software, USA)
Data source location	*KAIST, Daejeon 34141, Korea*
Data accessibility	*Data is provided within the article*

**Value of the data**

• This data shows that a Au nanowire injector (Au NWI) could be an useful tool for direct gene delivery into the pronucleus of mouse embryos.
• Negatively charged plasmids can be attached by electrostatic interaction and be detached by applying an electric pulse to a Au NWI.
• This data shows that a Au NWI can directly inject ~0.014 pg of the plasmid into a pronucleus of an embryo.
• This data presents that a Au NWI system does not induce detrimental damages on development of embryos and efficiently express the green fluorescence protein in vitro.

## 1 Data

A Au nanowire injector (Au NWI) directly delivered the transgene into pronucleus (PN) of 1-cell stage of an embryo. The Au NWI system does not affect detrimental damages on in vitro development of the mouse embryo. After a delivery of plasmid by Au NWI system, the successful expression of the green fluorescent protein (GFP) in M- or blastocyst(BL)-stage of the embryos was confirmed by confocal microscopy.

## 2 Experimental design, materials and methods

All experimental design, materials and methods were based on reported paper [1]. The scanning electron microscopy (SEM) images of the Au NWIs were obtained by FEI Nova 230 (EI Company, USA). The Au NWI injection was performed by a conventional microinjection system (Sutter instrument, USA) mounted on a Leica micromanipulator.

### 2.1 Schematic illustration of Au NWI-based plasmid delivery and optical image of a Au NWI injection setup

**Fig. 1:**
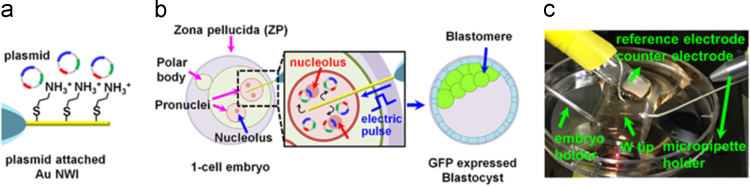
Schematic illustration of delivery of plasmid by a Au NWI. (a) Scheme of attachment of plasmid onto the surface of Au nanowire by electrostatic interaction between positively charged cysteamine and negatively charge of the plasmid. (b) Scheme of electro-triggered delivery of plasmid by a Au NWI. A Au NWI can penetrate the zona pellucida (ZP) and can precisely locate pronucleus (PN) of a 1-cell stage of embryo by passing the nucleoli in the PN. (c) An image of Au NWI injection setup for delivering plasmid into a mouse embryo.

To deliver plasmid from a Au NWI, the conventional microinjection system (Sutter) mounted on a Leica micromanipulator and an embryo holder pipette were used. The Au NWI was physically mounted on micropipette holder. First, a 1-cell stage of embryo was transferred in a culture dish containing M2 media without mineral oil. To release the plasmid from a Au NWI by applying an electric pulse, the Au NWI injection setup is composed of a 3-electrode system, a saturated calomel electrode (reference electrode), a Pt wire (counter electrode) and a Au NWI (working electrode) (Fig. 1).

### 2.2 Confirmation of attachment of plasmid onto a Au NWI by SEM-EDAX analysis

**Fig. 2:**
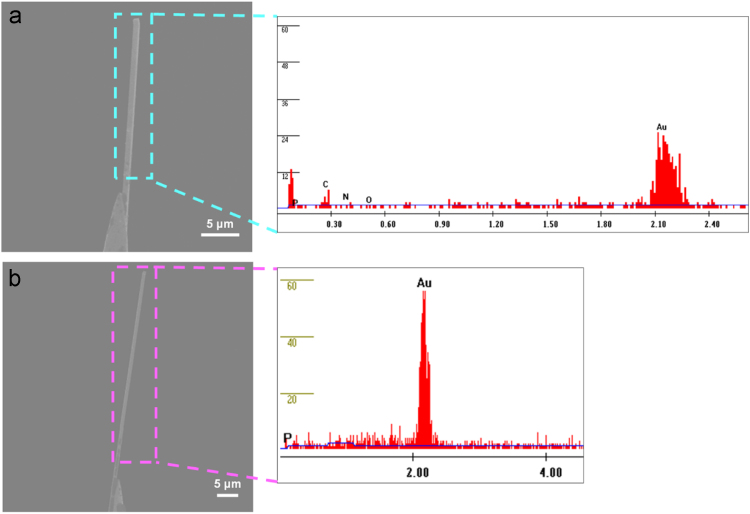
Scanning electron microscope (SEM) images of Au NWIs and Energy dispersive X-ray analysis (EDAX) spectra of Au NWIs. (a) In the case of plasmid-loaded Au NWI, the P peak appeared in EDAX spectra. (b) When the Au NWI was modified with only cysteamine, the P peak did not present in EDAX spectra.

To confirm the electrochemical attachment of the plasmid onto the Au NWI, 2 groups of Au NWI were prepared. First, the Au NWIs were incubated in 1 mL of 20 mM cysteamine(CA) for 30 min in ambient environment. Then, the Au NWIs were washed with distilled water to remove the excess CA. The CA-loaded Au NWIs were incubated in 700 μL of 100 nM phMGFP plasmid solution at room temperature for 10 h and the excess plasmid was washed with distilled water. As a control, the other Au NWIs were modified with only the CA via Au–S bond. After that, the scanning electron microscopy (SEM) images of Au NWIs were obtained (FEI, Nova 230) (Fig. 2). To confirm the attachment of phMGFP plasmids on the Au NWI, a phosphorus(P) peak originated from the backbone of a plasmid DNA was measured by EDAX-system attached to SEM (FEI, Nova 230). In the spectra, a phosphorus(P) peak was observed in the plasmid-loaded Au NWI (Fig. 2a). On the other hand, there was no P peak in only CA-loaded the Au NWI (Fig. 2b). This data indicated that plasmid attached successfully on the Au NWI via electrostatic interaction.

### 2.3 Detachment of plasmid from Au NWIs by applying an electric pulse

**Fig. 3:**
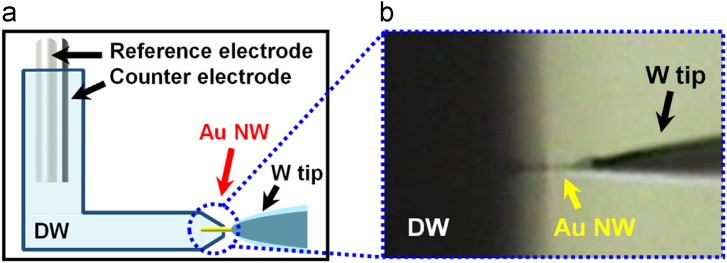
(a) Schematic illustration of the aqueous three-electrode system for the release of plasmids from Au NWIs via the application of an electric pulse. The system was constructed using a plasmid-loaded Au NWI (working electrode), a saturated calomel electrode (reference electrode), and a Pt wire (counter electrode). (b) Optical image of the immersion of a plasmid-loaded Au NWI into DW.

To electrically trigger the detachment of the plasmid from the Au NWIs, a −0.8 V of electric pulse was applied for 2 min to the Au NWI in distilled water (DW). To comprise a aqueous three-electrode system, a saturated calomel electrode (reference electrode), a Pt wire (working electrode) and a Au NWI (working electrode) were connected in DW filled home-made glass cell. Under microscopy, the only part of Au NW was dipped in DW (Fig.3). Immediately, an electric pulse was applied to Au NWI. After 2 min, the distilled water containing the plasmid was extracted and analyzed by PCR

### 2.4 Quantities of plasmid delivered into an embryo by Au NWI

**Fig. 4:**
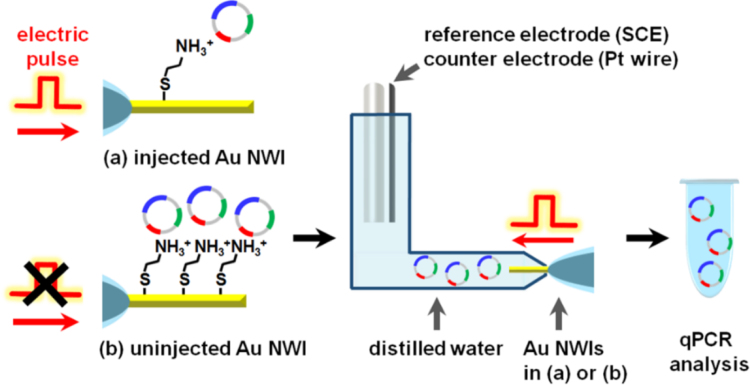
Schematic illustration of experimental design to examine the amount of delivered plasmid in 1-cell embryo by a Au NWI.

To quantify the amount of delivered plasmid by a single Au NWI injection, two types of Au NWIs were prepared; Fig. 4(a) injected Au NWIs which were used to inject and release the plasmid into a PN by an electric pulse, and Fig. 4(b) uninjected Au NWIs which were not used to inject the plasmid into an embryo. The plasmid remaining on the Au NWIs in (a) and (b) was released into DW by an electric pulse. After completion of the electric release, the DW with released plasmid from Au NWIs in (a) and (b) was collected and analyzed by the quantitative realtime PCR (qPCR), respectively.

**Fig. 5:**
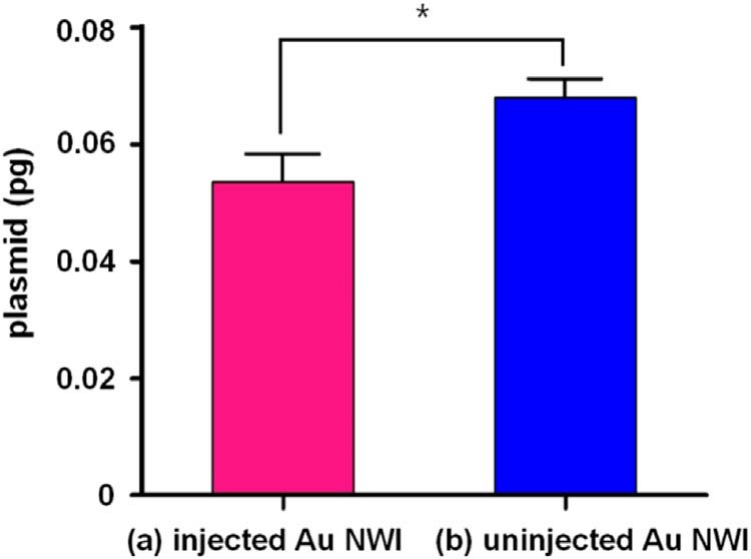
Amount of delivered plasmid (pg) by a Au NWI. Each quantity of plasmid from Au NWIs in (a) and (b) in Fig. 4 is shown; (a) injected Au NWIs which were used to inject and deliver plasmid (magenta) and (b) uninjected Au NWIs which were not used to deliver the plasmid (blue).

The qPCR analysis was performed using an ABI Prism 7000 sequence detection system (Applied Biosystems, USA) with SYBR Green PCR Master Mix (Applied Biosystems, USA). As a positive control, several concentrations of phMGFP (0.01, 0.05, 0.1, 0.5, and 1 ng) were prepared. Each test was performed in triplicate. The quantity of delivered plasmid was ~ 0.014 pg obtained by comparing Fig. 5(a) and (b). In each group, three different Au NWIs were used to qPCR analysis. *P < 0.05 versus (a) injected Au NWI.

### 2.5 Effect of Au NWIs injection on embryo development

**Fig. 6:**
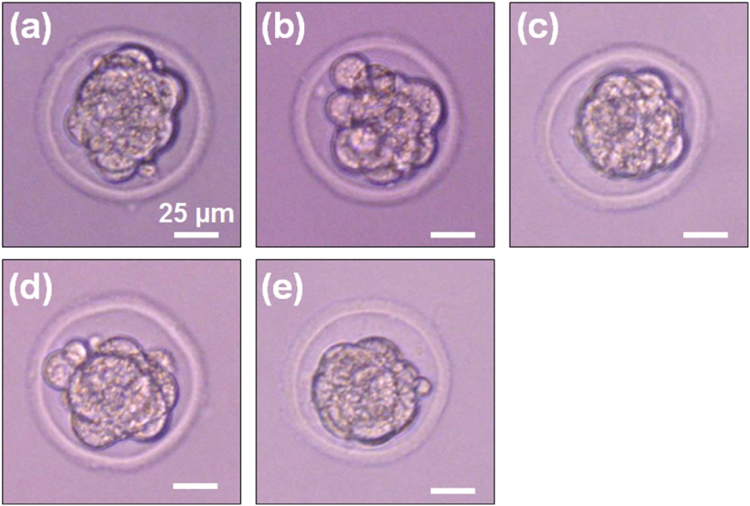
Optical images of morula(M)-stage embryos after 3 days of Au NWI injection. The diameters and lengths of the Au NWI are presented in Table 1. All embryos developed normally to the M stage.

To assess an effect of Au NWIs on embryos, 5 different Au NWIs having various diameter and length were tested (Table 1). The Au NWIs penetrated the ZP and injected into PN of the embryos. After removal of the Au NWIs, the embryos were individually incubated in M16 media covered with mineral oil for 3 days at 37 °C in an atmosphere containing 5% CO_2_. Fig. 6 shows that all embryos developed normally to the morula(M)-stage.

**Table 1:** Diameters and lengths of Au NWIs used to embryo viability test.

**# of Au NWI**	**Diameter (nm)**	**Length (μm)**
1	730	27
2	830	31
3	334	25
4	248	27
5	500	24

### 2.6 Effect of applying an electric pulse by using Au NWIs on 2-cell development of embryos

To assess an effect of applying an electric pulse by Au NWIs on embryos, 59 zygotes were injected by using Au NWIs with an electric pulse of −0.8 V for 2 min. After 2 min, the zygotes were incubated in M16 medium covered with mineral oil at 37 °C in an atmosphere containing 5% CO_2_. Next day, among 59 zygotes the 54 zygotes developed to 2-cell stage, indicating 92% rate of normal development. The number of embryos is shown in Table 2.

**Table 2:** Embryo development rate after Au NWI injection and applying an electric pulse.

**Total number of embryos**	**number of 2-cell embryos**	**number of degraded embryos**	**number of 1-cell bock embryos**	**Embryo viability rate**
59	54	2	3	92%

### 2.7 Confirmation of GFP expression by Au NWI-based gene delivery

To confirm the expression of green fluorescent proteins (GFP) in an embryo by Au NWI system, z-stack images of a morula (M)-stage of embryo was collected. By analyzing the Z-stack image, it was determined whether the embryo expressed the GFP in a third dimension. Since we delivered phMGFP plasmid [1], the GFP expressed when the plasmid is successfully integrated in genomic DNA of an embryo. The Moive 1 showed in a third dimensional expression of GFP in all blastomeres in a M-stage of the embryo, indicating no mosaicism. The M-stage of embryo was filmed at interval of 7.6 μm in Z-stack image by using a confocal microscope (Carl Zeiss, Germany).

## Figures and Tables

**Movie 1:**
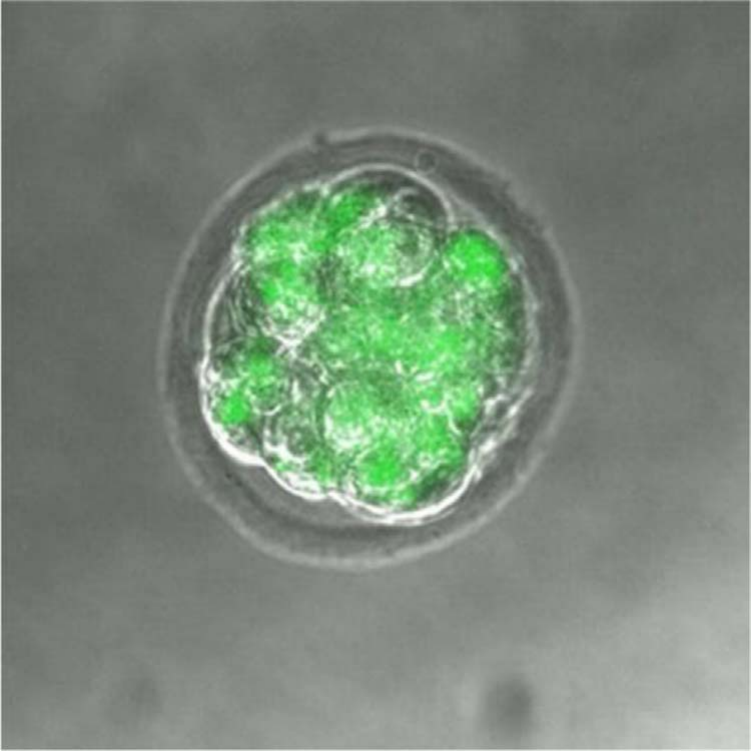
Representative z-stack movie of GFP-expression in a M-stage of embryo (without mosaicism) by delivering phMGFP plasmid via a Au NWI. Movie showed that transgene delivered by Au NWI expressed and produced the green fluorescent proteins (GFP) in all blastomeres.A video clip is available online.
